# Indehiscent sporangia enable the accumulation of local fern diversity at the Qinghai-Tibetan Plateau

**DOI:** 10.1186/1471-2148-12-158

**Published:** 2012-08-28

**Authors:** Li Wang, Harald Schneider, Zhiqiang Wu, Lijuan He, Xianchun Zhang, Qiaoping Xiang

**Affiliations:** 1 State Key Laboratory of Systematic and Evolutionary Botany, Institute of Botany, The Chinese Academy of Sciences, Beijing, 100093, China; 2 Department of Botany, Natural History Museum, London, United Kingdom; 3 Graduate University of Chinese Academy of Sciences, Beijing, China

**Keywords:** Chloroplast capture, Chloroplast DNA, Himalaya, Incongruent phylogenetic hypotheses, *Lepisorus clathratus*, Phylogenetic analyses, Reticulate evolution, Self-fertilization, Low-copy nuclear genes, *PgiC*, *LFY*

## Abstract

**Background:**

Indehiscent sporangia are reported for only a few of derived leptosporangiate ferns. Their evolution has been likely caused by conditions in which promotion of self-fertilization is an evolutionary advantageous strategy such as the colonization of isolated regions and responds to stressful habitat conditions. The *Lepisorus clathratus* complex provides the opportunity to test this hypothesis because these derived ferns include specimens with regular dehiscent and irregular indehiscent sporangia. The latter occurs preferably in well-defined regions in the Himalaya. Previous studies have shown evidence for multiple origins of indehiscent sporangia and the persistence of populations with indehiscent sporangia at extreme altitudinal ranges of the Qinghai-Tibetan Plateau (QTP).

**Results:**

Independent phylogenetic relationships reconstructed using DNA sequences of the uniparentally inherited chloroplast genome and two low-copy nuclear genes confirmed the hypothesis of multiple origins of indehiscent sporangia and the restriction of particular haplotypes to indehiscent sporangia populations in the Lhasa and Nyingchi regions of the QTP. In contrast, the Hengduan Mountains were characterized by high haplotype diversity and the occurrence of accessions with and without indehiscent sporangia. Evidence was found for polyploidy and reticulate evolution in this complex. The putative case of chloroplast capture in the Nyingchi populations provided further evidence for the promotion of isolated but persistent populations by indehiscent sporangia.

**Conclusions:**

The presented results confirmed the hypothesis that indehiscent sporangia promote the establishment of persistent population in different regions of the QTP. These results are consistent with the expectations of reproductive reassurance by promotion of self-fertilization that played a critical role in the assembly of populations in isolated locations and/or extreme habitats.

## Background

Several recent studies suggested that environmental condition enforced the evolution of self-fertilization in plants, especially as a strategy to adapt to extreme habitats [1] and along the trailing edge [2]. For example, evidence was reported for indirect selection of mating system during the evolution of drought resistance in the angiosperm genus *Mimulus* evolution [3]. These studies add alternative aspects to the well-established hypothesis of reproductive assurance as a cause for the establishment of self-fertilizing populations in plants [4]. Recent studies on the biology of fern dispersal, in particular long-distance dispersal emphasized evidence for a correlation of colonization of new locations and the capacity of self-fertilization [5-7].

The majority of ferns appear to reproduce either via out-crossing or mixed mating systems [6,8-11], but several mechanisms exist to promote inbreeding in ferns [12]. The catapult mechanisms of the sporangia, as found in the vast majority of species belonging to Polypodiales [13], are one of the most prominent features among derived ferns promoting dispersal [14] and subsequently out-crossing. However, a handful or so exceptions, which lack such catapult mechanisms, have been reported among derived ferns, e.g. some European spleenworts such as the tetraploid *Asplenium lepidum*[15,16] and the diploid *Asplenium jahandiezii*[17]. These sporangia are called throughout this paper ‘indehiscent sporangia’ whereas regular sporangia are called ‘dehiscent sporangia’. Very little is known about the origin of the evolutionary processes supporting the establishment of indehiscent sporangia. Here, we explore the hypothesis that indehiscent sporangia promote self-fertilization coinciding with geographical isolation, local survival, and adaptation to extreme environmental conditions.

To study this hypothesis, we explore the evolution of indehiscent sporangia in ferns occurring at the high altitudes of the Qinghai-Tibetan plateau (QTP), also called the “roof of the world”. Specimens with indehiscent sporangia belonging to the *Lepisorus clathratus* complex were first reported as the segregate genus *Platygyria*[18,19]. However, recent studies confirmed that these specimens belong to the *Lepisorus clathratus* complex [20-22]. Thus, this complex comprises not only specimens with regularly formed, dehiscent sporangia that open with the above mentioned catapult mechanism, but also specimens with irregularly formed, indehiscent sporangia. Besides, existing records show evidences for predominant occurrence of specimens with indehiscent sporangia at high altitudes >3,000 meter in contrast to specimens with dehiscent sporangia occurring at lower altitudes [19,22,23]. Indehiscent sporangia promoting self-fertilization may be an adaptation to the isolation of suitable habitats, extreme climatic windy conditions, and short vegetation periods in the extreme altitudes of the QTP. Consequently, several authors suggested an origin of *Platygyria*, = specimens with indehiscent sporangia, from *Lepisorus clathratus *complex, = specimens with dehiscent sporangia, during the uplift of the Himalaya [18,19,23]. Indehiscent sporangia are assumed to be advantageous to secure sexual reproduction, = reproductive assurance, in conditions of strong isolation of putative habitats. Thus, adaptive advantages of self-fertilization may explain multiple origins of indehiscent sporangia in the *Lepisorus clathratus* complex [22]. To avoid taxonomic confusion, the complex is treated as a single species, *L. clathratus*, throughout this paper. *Platygyria* is treated as a synonym of *Lepisorus*[20,21].

This study is carried out in the context of previously reported evidences suggesting multiple origins of indehiscent sporangia [22]. The previous report was based exclusively on maternally inherited chloroplast genome sequences (cpDNA) [24,25] and thus it was unable to account for introgression, reticulation, and polyploidy [26-28]. Polyploidy and the indication for reticulate evolution have been recorded for the genus *Lepisorus*[29] and the most recently also for the *L. clathratus* complex [30]. Thus, we explore the evolution of indehiscent sporangia by using sequence data of two cpDNA markers and two nuclear low-copy genes, *LFY* and *PgiC*, plus flow cytometry to determine the DNA ploidy level [31]. Finally, we expanded our sampling by including specimens with indehiscent sporangia collected in the Nyingchi prefecture, located in the southeastern part of Tibet Autonomous Region of China. The specimens will be named throughout this paper as Nyingchi *Platygyria*. The study is designed to provide a more comprehensive insight in the spatial-phylogeographic origin of indehiscent sporangia in the *Lepisorus clathratus* complex and the ecological-evolutionary consequences. Together with the Lhasa region, the Nyingchi region is known for the abundance of specimens with indehiscent sporangia compared to the Hengduan Mountains where specimens with indehiscent sporangia are less common than those with dehiscent sporangia.

## Methods

Samples were obtained via fieldwork by members of the research team in various parts of China and neighboring countries. Materials for DNA extraction were dried and stored in silica. Some plants were collected as living collections and cultivated at the conservatory of Institute of Botany, Chinese Academy of Sciences (IBCAS). Taxon sampling was guided by recently reported phylogenetic analyses [20-22] to include the resolved clades within *Lepisorus*, but particular care was taken to cover the geographic range of all morphologically distinct forms of *Platygyria*[19]. The two genera *Neocheiropteris* and *Tricholepidium* were sampled to have a glimpse of the phylogenetic relationships of *Lepisorus* and its allies.

Whole genomic DNA was extracted using a modified CTAB approach [32]. These DNA extracts were used to amplify two chloroplast regions *rps4-trnS* and *trnLtrnF* (consisting of *rps4* + *rps4trn*S intergenic spacer and *trnL* intron plus *trnLF* intergenic spacer) as described in Wang et al. [20]. The DNA was also used to amplify two nuclear genes, the low-copy gene *PgiC* using the primers and protocol of Ishikawa et al. [33] and the low-copy gene *LFY* using newly designed *LFY* primers for the introns located between exon1 to exon 3: FLFYE1dF: 5’-GGCAACGCCTRCAACTACT-3’; FLFYE3dR: 5’-CTTTGGYTTGTTGATRTACT-3’; FLFYE3eR: 5’-GCRTGTCGAAAAACYTGRTTGGT-3’. To separate the different allele copies, we performed cloning experiments using pGEM T-easy vectors (Promega Corp.) with a Pharmacia purification kit (Amersham Pharmacia Biotech) following the manufactures’ protocols. Four to seven clones of *LFY* gene were sequenced for the selected 12 specimens. Cloning and sequencing were applied on *PgiC* gene of 25 specimens (2–7 clones) which represented the key genotypes resolved in our previous study [22]. *PgiC* gene of the remaining 23 specimens was directly sequenced in order to reduce experimental expense. Sequencing reactions were carried out using the DYEnamic^TM^ ETDye Terminator Cycle Sequencing Kit (Amersham Pharmacia Biotech). Sequences were analyzed using MegaBACE^TM^1000 DNA Analysis Systems, following the manufacture’s protocols.

All newly generated sequences were assembled and edited using ContigExpress program from the Vector NTI Suite 6.0 (Informax Inc., North Bethesda, MD). BlastN searches were carried out for all newly generated sequences [34]. Alignments were generated using CLUSTAL X [35], and further adjusted manually in BioEdit [36] and MacClade 4.0 [37]. Ambiguously aligned regions were detected visually and excluded from further analyses. Only two small ambiguously aligned fragments were detected in the cpDNA (<3% of the total base pairs) sequences and none in the nuclear regions. Searches for sequence inversions were negative. For the two nuclear genes, we took into consideration of PCR bias, the putative occurrence of chimaeric sequences, and variation due to polymerase error by checking the sequences very carefully and blast sequences in NCBI ( http://www.ncbi.nlm.nih.gov). The copy numbers of the two nuclear genes (*PgiC* and *LFY*) in each specimen were not identified because the ploidy levels of most studied samples are uncertain. However, all analyses were carried out including all cloned sequences and with a reduced dataset including only sequences found in more than one clone per specimen. Besides newly generated sequences (all nuclear gene sequences and minority of chloroplast sequences), we used sequences that were generated for previous studies [20-22] and all newly generated sequences were deposited in GenBank (see Additional file 1). In total, we analyzed four datasets independently. The first dataset includes all cpDNA sequences, whereas the second and third datasets correspond to the two low-copy gene datasets *LFY* and *PgiC*, and the fourth dataset is constituted of the reduced *PgiC* sequences as mentioned above.

Gene-trees were reconstructed using standard phylogenetic methods such as maximum parsimony in PAUP 4.0b10 [38], maximum likelihood in Garli 1.0 [39] and PHYML 3.0 [40], and Bayesian inference in MrBayes 3.1.2 [41]. jModeltest 0.1.1 [42] was used to determine the model of sequence parameters for model-based approaches as maximum likelihood and Bayesian inference. Non-parametric bootstrap values were calculated for maximum parsimony with 1,000 bootstrap replicates and maximum likelihood with 500 replicates. All analyses were carried out using standard protocols as described in our previous studies [20-22] and the phylogenetic trees were visualized using FigTree 1.3.1. ( http://tree.bio.ed.ac.uk/software/figtree). Convergence of MCMC runs from Bayesian analyses were also studied using Tracer 1.5 ( http://tree.bio.ed.ac.uk/software/tracer). The burn-in phase was defined manually by studying carefully the output of TRACER. The estimated burn-in phase comprised fewer generations than the default 10% cut-off implemented in this software. Phylogenetic network analyses [43,44] were carried out using SPLITSTREE 4.10 ( http://www.splitstree.org) and DENDROSCOPE 3.0.13beta ( http://ab.inf.uni-tuebingen.de/softwar/dendroscope). These methods were used to visualize and infer alternative hypothesis of reticulate evolution in combination with established procedures (SH-test) and collapsing multiple trees (in DENDROSCOPE) and z-closure consensus networks (in SPLITSTREE). These methods were applied to distinguish between phylogenetic uncertainties and reticulate evolution; in particular we compared very carefully the signals given by different phylogenetic trees either visually in DENDROSCOPE or by calculating z-closure networks in SPLITSTREE. These analyses were carried out for three kinds of tree sets obtained by individual analyses of each of the three regions: (1) all most parsimonious trees recovered in maximum parsimony analyses, (2) all most likely trees recovered in maximum likelihood analyses, and (3) 100 bayesian trees recovered in Bayesian inference of phylogeny. Node support was further investigated using additional tests as implemented in PAUP, e.g. SH-test [45]. Regional diversity was evaluated by considering specimens and sequences used in this study and previous studies on the *Lepisorus clathratus* complex [20-22].

Four samples, representing the main distribution range of *L. clathratus* complex [22] and including the special “Nyingchi *Platygyria*”, were selected to explore ploidy levels because existing chromosome counts suggest the occurrence of diploids and tetraploids in the *L. clathratus* complex [30,46]. In the absence of materials suitable for additional chromosome counts, we employed flow cytometry [47,48] to determine the DNA ploidy level [31]. These values were determined using silica dried leaf material analyzed together with the internal standard consisting of leaf material of a specimen that was reported to be tetraploid [30]. The leaf material was chopped together with the internal standard, = tetraploid *Lepisorus clathratus* s.l., in 0.5 ml ice-cold general-purpose buffer with the addition of 3% PVP-40 [49]. The nuclear suspension was filtered through a nylon mesh and then incubated after adding a solution containing ribonuclease A (RNase A; Sigma-Aldrich, St. Louis, MI, USA). Finally, samples were stained with propidium iodide (Sigma-Aldrich, St. Louis, Missouri, USA) and incubated on ice for 30 minutes before analyzed using a BD FACSCalibur^TM^ flow cytometer (BD Bioscience, Franklin Lakes, NJ, USA). For each specimen, we counted about 40,000 nuclei per run. Only measurements were considered with a clear signal. The DNA ploidy level was recorded as relative to the internal tetraploid standard: < 4x, = 4x, > 4x.

## Results

**Figure 1:**
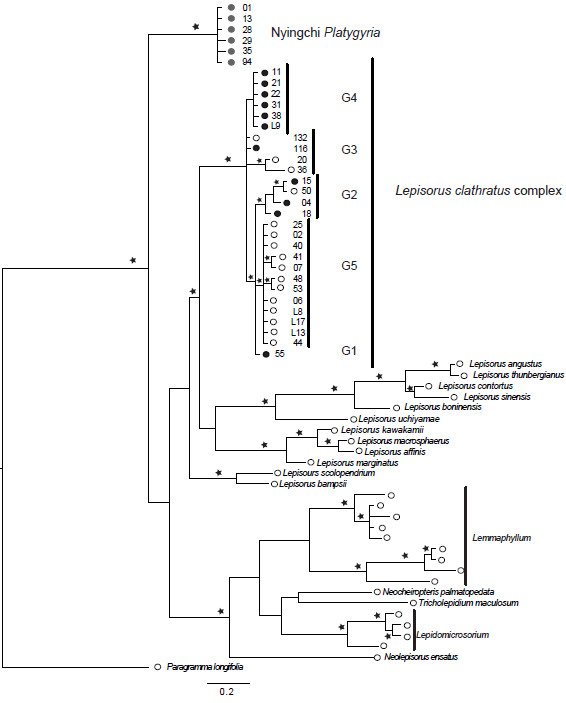
** Phylogeny of the cytoplasmic inherited chloroplast genome reconstructed using sequences of two marker regions.** The shown phylogeny is the mean consensus phylogram of the Bayesian inference of phylogeny carried out with partitioning coding versus non-coding regions. Stars indicate the Bayesian inference P-values ≥ 0.95. G1 to G5 correspond to the haplotype groups of the *Lepisorus clathratus* complex recognized in Wang et al. [22], whereas numbers indicate the collection number of the specimens of this complex and the Nyingchi *Platygyria* (grey circles). Circles indicate the type of sporangia: black and grey filled circles indicate specimens with indehiscent sporangia (= *Platygyria*), whereas open circles indicate specimens with dehiscent sporangia. Species names have been removed in the *Lemmaphyllum* and *Lepidomicrosorium* complex because their taxonomy is currently unresolved. Identical to nearly identical topologies (not shown) were recovered in maximum parsimony and maximum likelihood analyses of the same dataset.

Information concerning the total length of nucleotides, number of variable sites, number of parsimonious informative sites and the selected models by jModeltest [42] in three aligned datasets was given in Additional file 2. No chimaeric sequences were identified within the two nuclear gene datasets. Phylogenetic hypotheses obtained by phylogenetic analyses of the cpDNA sequence data recovered all specimens of *Platygyria* nested within the *Lepisorus clathratus* clade with the exception of the specimens of *Platygyria* collected in the Nyingchi region (Figures 1). These specimens, Nyingchi *Platygyria*, possessed a haplotype that was not found in any other species of the tribe Lepisoroideae. In fact, the cpDNA was not found to be part of the genus *Lepisorus* in the optimal ML, MP trees and in the consensus Bayesian phylogeny. Very little sequence variation, 99.5% identical base pairs, was found among the six specimens sampled in the Nyingchi region (Figures 1).

**Figure 2:**
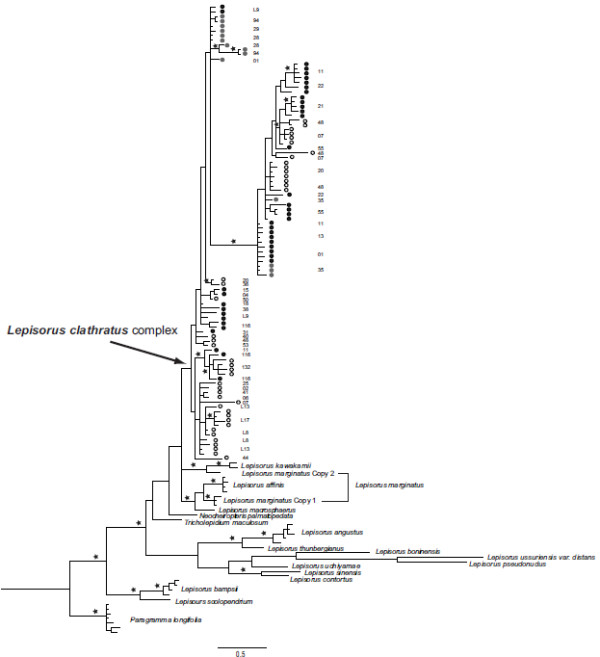
** Phylogeny of the nuclear genome as reconstructed using sequences of *PgiC *gene.** The shown mean consensus phylogram was obtained by carrying out a Bayesian inference of phylogeny with all obtained sequences, including all cloned sequences. Stars indicate Bayesian inference P-Values ≥ 0.95. Numbers indicate the collection number of the specimens. Circles indicate the type of sporangia: black and grey filled circles indicate specimens with indehiscent sporangia (= *Platygyria*), whereas open circles indicate specimens with dehiscent sporangia. Grey circles indicate Nyingchi *Platygyria*.The two distinct *PgiC* copies recovered in *Lepisorus marignatus* are marked as Copy 1 and Copy 2 plus a frame on the right side of the tree. Note: the Nyingchi *Platygyria* specimens are nested within the *Lepisorus clathratus* complex.

**Figure 3:**
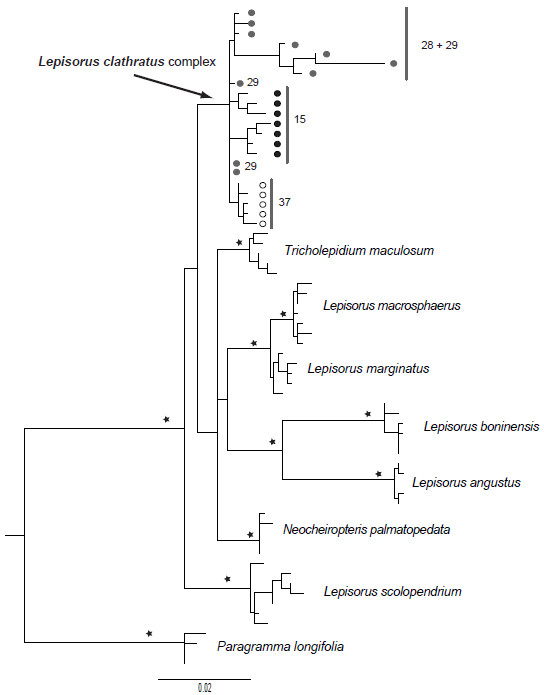
** Phylogeny of the nuclear genome as reconstructed using sequences of *LFY *gene.** Phylogram obtained via the maximum likelihood analyses of the *LFY* dataset including all sequences obtained via cloning. Stars indicate the maximum likelihood bootstrap support values ≥ 95%. Numbers indicate the collection number of the specimens. Circles indicate the type of sporangia: black and grey filled circles indicate specimens with indehiscent sporangia (= *Platygyria*), whereas open circles indicate specimens with dehiscent sporangia. Grey circles indicate Nyingchi *Platygyria.*

Phylogenetic hypotheses obtained by independent phylogenetic analyses of the two nuclear coding genes, *PgiC* (Figures 2) and *LFY* (Figures 3), found all specimens of *Platygyria* were nested within the *Lepisorus clathratus* complex clade, including also the Nyingchi *Platygyria* specimens.

**Figure 4:**
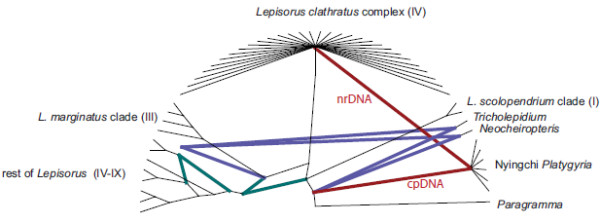
** Reticulogram obtained using Dendroscope based on the trees obtained from the three datasets (cpDNA, *LFY *and *PgiC *(full)).** Red lines mark the heritage of the cpDNA and nrDNA found in Nyingchi *Platygyria* specimens (conflicting relationships with the Bayesian inference P-values ≥ 0.95 in cpDNA, *LFY*, and *PgiC*). Blue lines illustrate the heritage of the cpDNA and nrDNA of the genera *Neocheiropteris* and *Tricholepidium* (conflicting relationships with the Bayesian inference P-values ≥ 0.95 in cpDNA, *LFY*, and *PgiC*). Green lines indicate conflicting evidence at the base of the *Lepisorus marginatus* and the rest of *Lepisorus* clades (conflicting relationships partly caused by putative hybrid origin of *L. marginatus* and partly caused by lack of robust relationships) The relationships among specimens of the *Lepisorus clathratus* clade were collapsed to simplify the figure. Roman numbers correspond to clade numbers given in Wang et al. [20].

According to SPLITSTREE and DENDROSCOPE analysis, the phylogenetic hypotheses obtained using cpDNA and nrDNA are incongruent (Figures 4). Firstly, the cpDNA separated the *Platygyria* specimens collected in the Nyingchi region from the *Lepisorus clathratus* complex clade, whereas the nrDNA recovers them as part of the *Lepisorus clathratus* complex clade (Figures 1, 2, 3). Secondly, the nrDNA did not support the division of *Lepisorus* versus the *Neocheiropteris*-*Tricholepidium* clade with the latter nested within *Lepisorus* in both *PgiC* and *LFY* based reconstructions (Figures 1, 2, 3). Thirdly, *Lepisorus marginatus* had two distinct copies of *PgiC*. One is found to be sister to *L. affinis* and the other sister to *L. kawakamii* (Figures 2). The first and third of these topological incongruences were recovered to be supported by bootstrap values and Bayesian posterior values (Figures 1, 2, 3) although the two nuclear genes lacked support for the deeper nodes. Thus, the second putative conflict may be the result of low resolution of the nuclear genes.The incongruence of the phylogenetic hypotheses recovered by cpDNA and two nrDNA markers were also supported by the SH-test, which found significant differences (p < 0.01) in the fitness of the topologies to the datasets: cpDNA = cpDNA based hypothesis is significantly better than the nrDNA based hypothesis; nrDNA = nrDNA based hypothesis is significantly better than the cpDNA based hypothesis.

**Figure 5:**
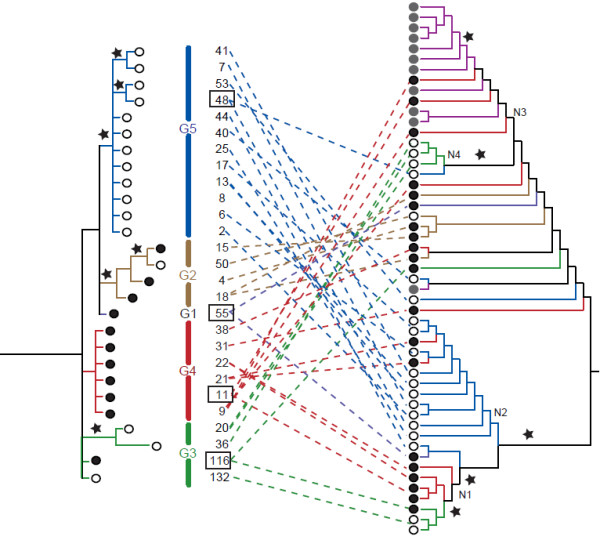
** Reticulate pattern observed by comparing the phylogeny obtained using the cpDNA (left) and *PgiC *(right).** Comparison based on the independent analyses of the cpDNA excluding Nyingchi *Platygyria* and *PgiC* data including Nyingchi *Platygryia* (for comparison see Figures 1 and 2). Numbers correspond to voucher numbers given in Additional file 1. Dotted lines connect cpDNA and *PgiC* copies of each specimen. Circles indicate the kind of sporangium: filled black circles = indehiscent sporangium with *L. clathratus* cpDNA, filled grey circles = indehiscent sporangium with non *L. clathratus* cpDNA (= Nyingchi *Platygyria*), open circles = dehiscent sporangium with *L. clathratus* cpDNA. Colors are chosen to visualize the correspondence of haplotype groups, G1-G5 as defined in Wang et al. [22] and *PgiC* copies. Squares indicate specimens with *PgiC* sequences belonging to highly divergent clades. Stars indicate the Bayesian inference P-values ≥ 0.95.

Within the *Lepisorus clathratus* complex, evidence for reticulation was recovered by visual comparison of the cpDNA and nrDNA (*PgiC*) based trees (Figures 5). The two recovered phylogenetic hypothesis are highly incongruent and several specimens show copies of *PgiC* belonging to different clades in the obtained phylogeny (Figures 5). Most specimens of haplotype group G5 showed *PgiC* sequences nested in a single clade (N2), but this was not the case for specimens of the haplotype groups G2, G3, G4. Specimens with cpDNA belonging to haplotype group G4 were found having very different *PgiC* sequences (clades N1 and N4). Highly heterozygotic specimens (Figures 5, *PgiC*) were found to be associated with different chloroplast haplotypes: specimen 11 with haplotype group G4, specimen 48 with haplotype G5, specimen 55 with haplotype group G1, and specimen 116 with haplotype group G3. All but one of the *PgiC* sequences obtained from Nyingchi *Platygyria* specimens (purple branches in Figures 5) were nested within one clade (N3) together with specimens sharing the G4 cpDNA (sister to a copy of *PgiC* obtained from specimen 40, Figures 5).

Specimens with indehiscent sporangia were found mainly in three regions (Table 1). These regions are distinct in the context of the frequency of specimens with dehiscent and indehiscent sporangia and the observed genetic variation of the chloroplast genome data. In two regions, the Lhasa region and the Nyingchi region, all studied specimens possessed indehiscent sporangia, whereas the Hengduan Mts showed a slightly higher frequency of specimens with dehiscent sporangia. The first two regions showed low cpDNA diversity and were dominated by specimens with cpDNAs belonging to one haplotype group. The Lhasa and Nyingchi regions had haplotypes that were exclusive to specimens with indehiscent sporangia occurring in this region. In contrast, most specimens with indehiscent sporangia collected in the Hengduan Mts shared the haplotypes recovered in specimens with dehiscent sporangia. This region showed high chloroplast genome diversity in both specimen groups (Table 1).

**Table 1:** Genetic variation of chloroplast genome of specimens with indehiscent sporangia for three core distribution regions

	Lhasa region *	Nyingchi region *	**Hengduan Mts.**
IS-DS	39 / 0	6 / 0	23 / 29
cpHG	3 / 0	1 / 0	3 / 4
cpHT	6 / 0	3 / 0	8 / 9

The table considers data presented in this study as well as previous studies [19,21]. IS-DS = Number of specimens with indehiscent versus specimens with dehiscent sporangia collected in this region. cpHG = cpDNA diversity measured by the number of haplotype groups. cpHT = cpDNA diversity measured by the number of haplotypes. The first number corresponds to the number of haplotypes found in specimens with indehiscent sporangia, whereas the second number represents the number of haplotypes found in specimens with dehiscent sporangia. * One haplotype group is exclusive or nearly exclusive to this region.

**Figure 6:**
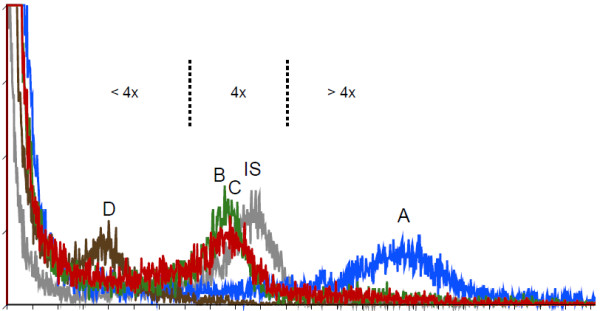
** Diagrams showing the result of flow cytometric determination of DNA ploidy levels of selected specimens.** A tetraploid sample belonging to the *Lepisorus clathratus* complex was used as the internal standard (IS, grey graph). X-axis shows the relative fluorescence, Y-axis the number of nuclei. **(A)***Platygyria* specimen 28 with cpDNA belonging to Nyingchi *Platygyria* (blue graph). **(B)***Platygyria* specimen 101with cpDNA belonging to G3 (green graph), **(C)***Platygyria* specimen 38 with cpDNA belonging to G4 (red graph). **(D)***Platygyria* specimen 119 with cpDNA belonging to G5 (brown graph). Dashed lines indicate the divide of the genome size interpretation: 4x, <4x, and >4x based on the IS = 4x.

Flow cytometry supported the hypothesis of different ploidy levels (Figures 6). Nyingchi *Platygyria* specimens had a higher DNA content in comparison to the tetraploid internal standard (> 4x for specimens 28) whereas other *Platygyria* specimens had either equal (= 4x, specimens 38 and 101, on the QTP) or lower (< 4x, specimen 119, Kangding in Hengduan Mts) DNA content in comparison to the tetraploid standard.

## Discussion

### Evidence for chloroplast capture

Strong conflicts were found between the phylogenetic hypotheses obtained using chloroplast and nuclear DNA sequence data (Figures 4). These conflicts can be sorted into two categories. The first category, conflicts such as the position of the genera *Neocheiropteris* and *Tricholepidium* may be the result of insufficient information or saturated variation in one or both datasets (Figures 4). This argument may especially apply to the highly variable regions used to reconstruct the phylogeny, as both *LFY* and *PgiC* markers are mainly based on intron data. Thus, the high substitution rate of these non-coding regions may show effects of sequence saturation. This argument is also consistent with the low support values recovered for the majority of the deep nodes using these markers (Figures 2, 3), but it also applies to the cpDNA (Figures 1). The second category, the conflict concerning the position of *Platygyria* specimens from the Nyingchi region, cannot be explained by these arguments. These specimens have *LFY* and *PgiC* sequences identical or nearly identical to specimens found in the *Lepisorus clathratus* clade (Figures 2, 3, 5), whereas the haplotypes of chloroplast sequences recovered in the Nyingchi *Platygyria* specimens are unique and have not been detected in any studied species of *Lepisorus* or related genera of lepisoroid or other ferns (Figures 1). In order to rule out any issue of contamination or related experimental errors, we performed additional independent DNA extractions, amplifications and sequencing of several specimens, using five specimens collected in the Nyingchi region and one collected between Maizhokunggar and Gongbo’gyamda located between Lhasa and Nyingchi. .

The cpDNA phylogeny suggests a long time isolation of this chloroplast type found in the Nyingchi specimens (Figures 1) and thus the results are not compatible with the hypothesis of incomplete lineage sorting. In the maximum parsimony analyses, they were recovered as sister lineage to *Paragramma*, but this may be the result of long-branch attraction. Our results of the cpDNA and nrDNA data suggest independent inheritance of the two genomes. They are also contrast with other collections of *Platygyria* (with indehiscent sporangia), which have both cpDNA and nrDNA nested within the *Lepisorus clathratus* clade (Figures 1, 2, 3).

The hypothesis of chloroplast capture provides the most likely explanation for the recovered conflict between the phylogenetic results of the cpDNA and nrDNA inheritance. The origin of the Nyingchi genotype is assumed to involve initial hybridization between individuals of the *Platygyria* type and an unknown species of *Lepisorus*. In this scenario, this unknown parent contributed the chloroplast because the cpDNA is maternally inherited in ferns [24,25]. Consequently, the nuclear genome is inherited paternally as required in processes involving chloroplast capture [50,51]. Remarkably, the cpDNA of the Nyingchi *Platygyria* specimens has not been recorded in any species of *Lepisorus* and relatives despite a rather comprehensive sampling in previous studies [20,21]. Morphological studies do not suggest any unique species of *Lepisorus* or its relatives in Nyingchi region. However, previous studies provided evidence that the reproduction with indehiscent sporangia, can result in populations with locality-persistent occurrences for considerably long time [22]. Thus, the combination of geographic isolation and indehiscent sporangia may have contributed to the origin and fixation of the Nyingchi *Platygyria* genotype.

The presented results are only the second report of putative chloroplast capture in ferns [52]. However, this may not necessarily indicate that the probability of chloroplast capture in ferns is low because only a small number of phylogenetic studies on ferns have integrated both chloroplast and low-copy nuclear genes so far [53-59].

### Evolution of indehiscent sporangia

Similar to the cpDNA, indehiscent sporangia are not associated with a single copy or clade of copies of *PgiC* (Figures 2), which provided further evidences for multiple origin of indehiscent sporangia. However, the pattern is also consistent with the hypothesis of indehiscent sporangia caused by mutations through recombination, introgression or hybridization between specimens with and without indehiscent sporangia. The above discussed chloroplast capture provided further evidence to this hypothesis of introgression of specimens with and without indehiscent sporangia. In addition, this study provides evidences for ploidy level changes, which supports multiple origins via hybridization combined with polyploidy as the most likely scenario although one specimen (no. 119) of *Platygyria* was found to be probably a diploid (see Figures 6). The argument on recombination is based on the reproductive biology of specimens with indehiscent sporangia (*Platygyria* type). Sporangia without the catapult mechanisms promote self-fertilization among the gametophytes developed from spores formed by meiosis events within a single sporangium. Actually, the mechanism enhances the probability that egg cells of archegonia of these gametophytes are fertilized by sperm cells developed in antheridia formed by gametophytes originated from the same sporangium. Thus, the self-fertilization may be either inter-gametophytic or intra-gametophytic [13]. At the same time, the sperm cells of gametophytes formed from spores originated within a single indehiscent sporangium may be able to fertilize gametophytes of individuals shedding their spores via dehiscent sporangia. Thus, the character of indehiscent sporangia may be inherited by zygotes that are formed by the fertilization of egg cells of gametophytes originated from sporophytes with dehiscent sporangia and sperm cells of gametophytes originated from sporophytes with indehiscent sporangia. This hypothesis proposes a linkage between paternal inheritance and indehiscent sporangia. The above discussed evidence for chloroplast capture supports this hypothesis because cpDNA is maternally inherited whereas the nuclear DNA is paternally inherited.

Previous studies on the allotetraploid *Asplenium lepidum*[15,16] have provided evidence for the origin of taxa with indehiscent sporangia via hybridization and polyploidy. Our results are consistent with the hypothesis that the origin of indehiscent sporangia involves these processes. However, the current estimates of ploidy levels are insufficient to prove that the reticulate pattern observed between the cpDNA and nrDNA is the result of hybridization and polyploidy, not caused by recombination among specimens with the same ploidy level.

However, the establishment of indehiscent sporangia is not necessarily linked to these processes as illustrated by the diploid *Asplenium jahandiezii*[17]. This taxon is endemic to a small region in southeastern France, the Gorge du Verdon. The sister species, *A. bourgaei* is also diploid but distinct by dehiscent sporangia and an occurrence in the eastern Mediterranean [18]. Both species are considered Tertiary relicts and current range of *Asplenium jahandiezii* is part of a region considered as Pleistocene glacial refugia of ferns and other plants [60-62]. Thus, indehiscent sporangia may have successfully promoted local survival of this species in a rather small and well-defined region of southeastern France.

Local survival through the last glacial maximum has also been discussed for occurrences of *Lepisorus clathratus* complex with indehiscent sporangia (*Platygyria*) at the QTP [22]. The newly discovered evidence for an isolated taxon in the Nyingchi region, Nyingchi *Platygyria*, adds a further support for the emerging hypothesis that self-fertilization promotes persistence of populations despite isolation and environmental challenges [1,2].

### Consequences for taxonomic research

This study sheds new light on the difficult taxonomy of the *Lepisorus clathratus* complex and in particular the treatment of *Platygyria*. The latter was recently reduced to be a synonym of *Lepisorus*[20]. Here, we found further support that the majority of *Platygyria* genotypes are intermingled with *Lepiosorus clathratus* genotypes [20,22]. However, the new results suggesting different ploidy levels support the hypothesis that the complex comprises at least two diploid entities that contributed to the origin of polyploids via hybridization. Future studies will need to focus on the untangling of the reticulate evolution that may be comparable to complexes such as *Asplenium lepidum* and its diploid parents, *A. aegeum* and *A. dolomiticum*[15,16].

Without doubt, the specimens treated here as Nyingchi *Platygyria* form a separate taxonomic entity. Future work will need to address the taxonomic implications of this study, such as the identification of the species name for these specimens from several names established for species with indehiscent sporangia [18,19].

### Assembly of plant diversity on the roof-of-the-world

In the recent years, many studies addressed aspects of the evolution of plants on the roof-of-the-world. Some of these studies resemble our own results in pointing out the remarkable evolution of unusual traits in adaptation to the alpine environments such as colored bracts [63] and the cushion growth forms [64] in various genera of eudicots. In our opinion, indehiscent sporangia may be such a selective advantageous character.

The report of the Nyingchi area as a putative mixture zone and survival area is also highly relevant to the current discussion on the history of plant diversity at this region in the last 4–5 million years [65]. In particular, the documentation of the Nyingchi area as survival area (refugia) is consistent with results on other plants such as *Cupressus* and *Mecanopsis*[66,67]. Furthermore, our result resembles the study on *Mecanopsis* in the documentation of the establishment of hybrid taxa in isolated locations along the southern border of the species range [67]. Establishment of polyploid hybrids appears to be a rather common process in the response of alpine plants to glacial cycles not only in the Himalaya but also in other areas such as the European Alps [68].

## Conclusions

The study found evidence that indehiscent sporangia promote the persistence of circumstances despite isolation and challenging climatic conditions in the Himalaya. Some of the evidence was based on the observation of a unique chloroplast genome in specimens with indehiscent sporangia collected in the isolated Nyingchi region. The origin of this chloroplast is best explained by chloroplast capture. Future research needs to focus on two core approaches: 1) additional data are required to reconstruct the contribution of reticulation and polyploidy; 2) sampling of data should allow reconstructing the population history of these taxa in three regions discussed: Hengduan Mts, Lhasa and Nyingchi regions. In general, these results raise questions concerning the contribution of shifts in the mating system to the origin of the unique plant diversity of the Himalaya.

## Competing interests

The authors declare no competing interests.

## Authors’ contributions

QPX, ZXC and HS designed the study. WL and ZXC collected materials. HLJ, WL and WZQ finished molecular experiments. WL and HS analyzed data. WL, XPQ, ZXC, and HS wrote the manuscript. All authors read and approved the final manuscript.

## Supplementary Material

Additional file 1 **Information regarding collection number, taxon names, collecting localities, voucher number and Genbank accession numbers.** IS: indehiscent sporangial type; DS: dehiscent sporangial type. Specimens utilized for ploidy level estimation are also included in the file, and the signs “A, B, C, D and IS” added after the collection number of them are corresponding to the signs used in Figures 6. Click here for file

Additional file 2 **Information concerning the length of nucleotides, the number of variable sites, the number of parsimonious informative sites and the models selected by jModeltest in the three aligned datasets (cpDNA, *LFY *and *PgiC*(full)).** Also, the percentages of variable sites and parsimonious informative sites in the total base pairs are also given. The cpDNA dataset indicates the combined dataset of *rps4-trnS* and *trnL-F*. Click here for file
